# Does the perception of HIV risk among Female sex workers affect HIV prevention behavior? application of the Health Belief Model (HBM)

**DOI:** 10.1186/s12889-022-14046-3

**Published:** 2022-08-30

**Authors:** Adane Asefa, Gachana Midaksa, Qaro Qanche, Wondimagegn Wondimu, Tadesse Nigussie, Biruk Bogale, Frehiwot Birhanu, Zufan Asaye, Nuredin Mohammed, Tewodros Yosef

**Affiliations:** 1 School of Public Health, College of Medicine and Health Sciences, Mizan-Tepi University, Mizan Aman, Ethiopia; 2 Department of Public Health, College of Health Science, Salale University, Salale, Ethiopia; 3 Department of Statistics, College of Natural and Computational Science, Mizan-Tepi University, Tepi, Ethiopia; 4 Department of Nursing, College of Medicine and Health Science, Mizan Tepi University, Mizan Aman, Ethiopia

**Keywords:** FSW, HBM, HIV/AIDS, Ethiopia

## Abstract

**Background:**

High prevalence of Human Immune virus/Acquired immunodeficiency syndrome (HIV/AIDS) in Female Sex Workers (FSWs) is identified as a bottleneck in fighting against HIV/AIDS. To this end, the international community planned a strategy of 'Ending inequality' and 'Ending the AIDS epidemic' by 2030. This could not be achieved without due attention to FSWs. Thus, this study attempted to assess HIV prevention behavior and associated factors among FSWs in Dima district of Gambella region, Ethiopia by using the Health Belief Model.

**Methods:**

A community-based cross-sectional study was conducted from March to May 2019 among 449 FSWs selected using the snowball sampling technique. Socio-demographic features, knowledge about HIV, attitude toward HIV prevention methods, and Health Belief Model (HBM) constructs (perceived susceptibility to and severity of HIV, perceived barriers, and benefits of performing the recommended HIV prevention methods, self-efficacy, and cues to practice HIV prevention methods) were collected using face to face interview. Data were entered into Epi-data 3.1 and analyzed using SPSS version 23. Bivariable and multivariable binary logistic regression analysis was done to identify the association between dependent and independent variables. *P*-value < 5% with 95 CI was used as a cutoff point to decide statistical significance of independent variables.

**Results:**

In this study, 449 FSWs participated making a response rate of 98.90%. Of these, 64.8% had high HIV prevention behavior. Age (AOR = 1.911, 95% CI: 1.100, 3.320), knowledge of HIV (AOR = 1.632, 95% CI: 1.083, 2.458), attitude towards HIV prevention methods (AOR = 2.335, 95% CI: 1.547, 3.523), perceived barriers (AOR = .627, 95% CI: .423, .930), and self-efficacy (AOR = 1.667, 95% CI: 1.107, 2.511) were significantly associated with high HIV prevention behavior.

**Conclusion:**

The study identified that about two third of FSWs practiced the recommended HIV prevention methods. Age of respondents, knowledge of HIV, favorable attitude towards the recommended HIV prevention methods, high self-efficacy, and low perceived barrier were associated with high HIV prevention behavior. Therefore, focusing on these factors would be instrumental for improving effectiveness of the ongoing HIV prevention efforts and attaining the 'Sustainable Development Goals of 'Ending inequality' and 'Ending the AIDS epidemic' by 2030.

## Background

Despite tremendous efforts, HIV/AIDS cases are not decreasing as expected and continue to cause a high number of deaths [1]. In 2019, 1.7 million people newly acquired HIV infection worldwide and 700, 000 died of AIDS-related causes [1, 2]. At the end of 2020, the number of people living with HIV/AIDS in the world reached 12 million [1, 3]. In Sub-Saharan Africa, adolescent girls and young women account for one out of four HIV infections [2, 4, 5]. In Ethiopia, more than 665,723 people are living with HIV, with the highest prevalence in the Gambella region (4.8%) [6]. In addition, 17.32% of the country’s expenditure for infectious and parasitic diseases went to HIV/AIDS [7].

In 2019, the majority (62%) of global new adult HIV infections were among key populations, including sex workers and their sexual partners [1–3]. Female sex workers (FSWs) are people who identify as female and who receive money or goods in exchange for sexual services, either regularly or occasionally, and who consciously define those activities as a source of income even if they do not consider sex work as their occupation [8]. FSWs accounted for an estimated 8% of the population worldwide and 5% in Eastern and Southern Africa [2]. There were an estimated 220,623 FSWs in Ethiopia in 2020 [7]. The risk of acquiring HIV among this segment of population is about 30 times higher than the general population [1, 3, 7, 9].

Various factors contribute to the susceptibility of sex workers to HIV. These include multiple and non-regular partners, more frequent sexual intercourses, unsafe working conditions, barriers to the negotiation of consistent condom use, limited access to services, and little control over these factors due to stigma and discrimination [7, 8].

To solve this, the United Nations Program on HIV/AIDS (UNAIDS) developed an ambitious goal to end HIV/AIDS epidemic as a public health threat by 2030 [9]. Accordingly, up to 2019, more than 40 countries made significant progress toward the HIV prevention targets of 2020 [1]. Despite this, HIV prevention efforts are not currently having the impact required to end AIDS by 2030. Tens of millions of people at risk of the virus are still not able to benefit from HIV prevention and health-protecting and life-saving interventions [1, 3]. In addition, the estimated 1.7 million people newly infected with HIV in 2019 were more than three times the 2020 target [1, 2].

As part of the sustainable development goal (SDG), the international community planned to reduce the number of new HIV infections and AIDS-related deaths to less than 370,000 and 250,000 respectively by 2025 [1, 3]. However, this is inevitably questionable without due emphasis on sex workers [1, 3]. The goal could further deteriorate with the COVID-19 pandemic [1, 2].

Sex workers can substantially reduce the risk of HIV transmission from clients and to clients through consistent and correct condom use, testing for HIV, and not sharing injecting equipment with others [3]. Each person is important for promoting these behaviors, which intern is affected by an individual’s beliefs, values, tendencies, and habits [10].

A qualitative study from a similar setting explored contextual factors embedded in the community that contributed to the high prevalence of HIV like practices of extramarital sex, polygamy, high movements of people to and from the area, and the existence of a large number of FSWs [11]. As a result, it is imperative to assess sex workers’ beliefs about HIV and their motivation to engage in prevention behaviors.

### Overview of Health Belief Model (HBM)

This model was developed by Rosenstock et al. to investigate the widespread failure of people to undertake preventive health measures [12, 13]. Since then, the HBM is one of the most widely employed models in health behavior interventions with above 60% average predictive capability [12]. The model elaborated on the reasons why certain people take actions that aim at preventing diseases while others avoid such actions [12]. HBM hypothesizes that an individual’s engagement in a healthy behavior is the function of his/her perception of the threat (susceptibility to the disease and perceived severity of the disease); behavioral evaluation (perceived benefit of undertaking the behavior and perceived barrier to the behavior); cues to perform the behaviors; and self-efficacy of successfully carrying out the indicated behavior [12, 13].

HBM has been successfully used for predicting and explaining a wide range of behaviors in different health researches [14–16]. Though it is one of the hotspot areas for HIV, there is limited evidence of HIV prevention behaviors among FSWs in Dima district. Therefore, this study aimed at assessing HIV prevention behaviors among FSWs in the District, using HBM. The findings from the study will be used for informing policymakers and planners to formulate HIV prevention strategies for reducing HIV among FSWs.

## Method and material

### Study design, setting, and period

A Community-based cross-sectional study was conducted from March to May 2019 in the Dima district. The district is found in the Agnuak zone of Gambella regional state at 628 km far from Addis Ababa, the capital city of Ethiopia, in the southwest direction. It was purposely selected considering the high prevalence of HIV/AIDS in the zone. There are about ten mining centers in the district. As a result, there are several gold mineworkers and female sex workers in the area. Clients of FSWs are frequently miners in this area who are reported to engage in risky sexual practices and have high prevalence of HIV [11, 17]. This study is part of the study conducted in the Dima district of Gambella region regarding HIV prevention behaviors among gold mineworkers [17].

### Study population

The source population for this study was all female sex workers in the Dima district. FSWs who selected through snowball sampling were the study population. FSWs stayed in the area for more than six months were included in the study while those FSWs who were severely ill or unable to communicate verbally during the time of data collection were excluded from the study.

### Sample size determination, sampling technique

The sample size was calculated with single population proportion formula by considering; 61.1%, the proportion of FSWs practicing HIV prevention methods from a study done in the Afar region, Ethiopia [18], 95% Confidence level, 4.7% margin of error, and 10% non-response rate. Finally, the calculated sample size was 454.

The snowball sampling technique was used to recruit study participants. The initial information was taken from the index sex workers who had been registered at the district health office and given counseling and reproductive health services. After getting the first participants, discussions were made with them to indicate other female sex worker(s) peers who can participate in the study. A similar procedure was followed until the desired sample size was fulfilled.

### Study variables, data collection tools, and measurement

The outcome variable was HIV prevention behaviors that measured using three “yes/no” response items; 1) Have you been using condoms consistently during each sex for the last six months? 2) Have you ever shared injecting equipment with anyone else in the last six months of the study? 3) Did you test for HIV infection in the last three months? Finally, the sex worker was categorized in high HIV prevention behavior if they answered “yes” for the first and third items, and “no” for the second item [18, 19]. These are the only indicators used as HIV prevention behavior for FSWs, provided that they can neither refrain from sexual contact nor live with a single sexual partner.

Independent variables comprised socio-demographic characteristics (Age, marital status, religion, educational status, place of residence), alcohol use, khat chewing, knowledge of HIV, HBM constructs (perceived susceptibility, perceived severity, perceived benefit, perceived barrier, cue to prevention behaviors, and self-efficacy).

#### Knowledge

Seventeen items that responded on yes/no were used to measure knowledge of HIV. Then, the mean score was computed, and participants who scored greater than the mean score of knowledge questions were categorized as having a high knowledge of HIV [20].

#### Attitude toward HIV prevention methods

Assessed by thirteen items on five points Likert scale ranging from 1 to 5 (1 strongly agree to 5 strongly disagree). The mean score was computed after reverse coding was done for negatively stated items. Participants who scored greater than the mean score were categorized as having a favorable attitude toward the aforementioned HIV prevention measures [20].

#### Perceived susceptibility

Is defined as an individual’s beliefs about the chances of contracting HIV/AIDS and related problems [12, 13, 21]. Five items were used to measure this construct on five points Likert scale. The composite score of the items was calculated and interpreted as high perceived susceptibility if the score was greater than the mean score.

#### Perceived severity

This refers to one’s beliefs of how serious HIV/AIDS and its consequences are. It implies that people must perceive HIV/AIDS as a serious infection that has severe consequences on their physical and social lives before they would adopt prevention behaviors against HIV/AIDS infection [12, 13, 21]. This variable was measured by six items on five points Likert scale. The composite score of the items was calculated and interpreted as high perceived severity if the score was greater than the mean score.

#### Perceived benefit

Refer to one’s beliefs in the efficacy of the recommended HIV preventative behaviors (consistent condom use, testing for HIV, and not sharing materials with others) in reducing the perceived severity of HIV/AIDS [12, 13, 21]. This construct was measured by seven items on five points Likert scale. The composite score of the items was calculated and interpreted as a high perceived benefit if the score was greater than the mean score.

#### Perceived barrier

Perceived barriers refer to one’s belief about tangible and psychological costs of the recommended HIV prevention behaviors, including phobic reactions, physical as well as psychological barriers, accessibility factors, and personal characteristics like inconveniences and unpleasantness to engage in HIV prevention behavior [12, 13, 21]. Seven items were used to measure this construct on five points Likert scale. The composite score was calculated and labeled as having a high perceived barrier if the score was greater than the mean score.

#### Cue to action

Cues to action are events or experiences, people, and media publicity that motivate a person to engage in HIV prevention activities. Cues to action are when an individual feels the desire to take the action after believing that one can do so [12, 13, 21]. This construct was measured by four items on five points Likert scale. The composite score was calculated and labeled as having a high cues to action if the score was greater than the mean score.

#### Self-efficacy/competency

Perceived self-efficacy refers to people’s judgment of their ability to organize and execute HIV prevention activities. Such expectations of personal efficacy determine whether the behaviors will be initiated, how much effort will be spent, and how long they will be sustained in the face of barriers and adverse experiences [12, 13, 21]. Seven items were used to measure this construct. Th composite scores of items was calculated and interpreted as a high self-efficacy if the score was greater than the mean score.

### Data quality control

The data were collected using a standard structured questionnaire for HBM constructs [12, 21] adjusted for HIV prevention guidelines for sex workers [1, 2, 6]. The English version questionnaires were translated to the local language “Amharic language” for data collection. Data were collected by trained ten data collectors and three supervisors qualified with Bachelor of Science (BSc) in Nursing. The collected data were evaluated for completeness, clarity, and consistency by the supervisor and investigators daily.

### Data processing and analysis

The collected data were coded and entered into Epi-Data manager version 4.0.2.101 cleaned and analyzed using SPSS version 23 statistical software. Descriptive statistics such as frequencies, means, standard deviation (SD), and proportions were done for different variables. Bivariable binary logistic regression analysis was done to select candidate variables at *p* values less than 25% for multivariable binary logistic regression. A *p-*value of less than 5% was used to declare the level of significance for HIV prevention behavior in the multivariable binary logistic regression.

## Results

### Socio-demographic characteristics

A total of 449 participated in this study with a 98.90% response rate. The mean age of the study participants was 21.84 (SD = 4.25) and 179 (39.9%) were in the age range of 16–19 years. Slightly above half; 242(53.9%) of the respondents were orthodox in religion whereas more than three fourth; 384(85.5%) of them were single. Respondents who reported primary education as the highest level attained accounted for 326(72.6%) while 87(18.4%) attended secondary education and above. All most all respondents, 433 (96.4%) served one customer a day. More than nine in every ten respondents; 414 (92.2%) had no other source of income (Table 1).

**Table 1:** Socio demographic characteristics of FSWs in Dima district, Southwest Ethiopia; 2019 (*N* = 449)

Variables	Response	Frequency	Percent (%)
Age	16–19	179	39.9
	20–24	165	36.7
	> = 25	105	23.4
Religion	Orthodox	242	53.9
	Muslim	100	22.3
	Protestant	100	22.3
	Others	7	1.6
Marital status	Single	384	85.5
	Married	10	2.2
	Divorced	52	11.6
	Widowed	3	0.7
Educational status	No formal education	36	8
	Primary Education	326	72.6
	Secondary Education or above	87	18.4
Number of Costumers served a day	1	433	96.4
	2	16	3.6
Additional source of income	Yes	35	7.8
	No	414	92.2
Additional income generating type of work(*n* = 35)	Waitress	16	45.7
	Daily labor	13	37.1
	Others	6	17.1

### Knowledge, attitude, and behavior related factors

Of the total respondents, 351(78.2%) were alcohol drinkers of whom, 211 (47%), always drink alcohol before sex. Similarly, 215 (47.9%) respondents were khat chewers. Of these, 186 (41.4%) were chewing khat during the survey, and 113 (25.2%) were doing so daily. Of the interviewed respondents, 287(63.9%) and 225 (50.1%) had high knowledge of HIV and a favorable attitude toward the recommended HIV prevention methods respectively (Table 2).

**Table 2:** Knowledge, Attitude and Behavior Related factors of FSWs in Dima district, Southwest Ethiopia; 2019 (*N* = 449)

Variables	Response	Frequency	Percent (%)
Alcohol Drinking	Yes	351	78.2
	No	98	21.8
Alcohol drinking before sex (*n* = 351)	Yes	211	47.0
	No	140	31.2
Pattern of alcohol use before sex (*n* = 211)	Always	72	47.0
	Sometimes	127	28.5
	Causal	12	2.7
Khat chewing	Yes	215	47.9
	No	234	52.1
Currently chewing khat (*n* = 215)	Yes	186	41.4
	No	29	6.5
Number of khat chewing in a week (*n* = 215)	Daily	113	25.2
	Every other day	51	11.4
	Twice a week	18	4.0
	Once a week	33	7.3
Knowledge of HIV (*n* = 449)	high knowledge	287	63.9
	low knowledge	162	36.1
Attitude toward HIV Prevention methods (*n* = 449)	Favorable attitude	225	50.1
	Un favorable attitude	224	49.9

### HIV prevention behaviors and related characteristics of FSWs

Out of the total 449 respondents, almost all; 428 (95.3%) had ever tested for HIV in their life, and about three fourth; 341 (75.9%) had tested in the past three months before the study. In addition, 285(63.5%) respondents consistently used a condom during each sex for the last six months before the study. Similarly, the majority of them; 384 (85.5%) were not sharing any injecting equipment with another person, in the past six months before the study. Accordingly, about two third; 291 (64.8%) (95 CI: 60% to 69%), of the respondents had high HIV prevention behavior, which was obtained by counting “yes” from the first and third items, and “no” from the second item (Table 3).

**Table 3:** HIV prevention behavior among FSWs in Dima district, Southwest Ethiopia, 2019 (*N* = 449)

HIV prevention methods	Responses	Frequency	Percent (%)
Ever tested for HIV	Yes	428	95.3
	No	21	4.7
tested for HIV in the past three months (*n* = 428)	Yes	341	75.9
	No	87	19.4
Consistent condom use in the past six months	Yes	285	63.5
	No	164	36.5
The pattern of condom use	Always	285	63.5
	Sometime	129	28.7
	Causal	24	5.3
	Never	11	2.4
Sharing of injecting equipment with others in the past six months	No	384	85.5
	Yes	65	14.5
HIV prevention behavior	high prevention behavior	291	64.8
	low prevention behavior	158	35.2

Pearson correlations were performed between all HBM constructs and HIV prevention behavior. Accordingly, the perceived barrier to HIV prevention was significantly and negatively correlated to HIV prevention behavior (*r* = -0.147, *p* < 0.001). However, self-efficacy was significantly and positively correlated to the prevention behavior(*r* = -0.097, *p* < 0.05). Similarly, except for perceived benefit and perceived barrier, all other HBM constructs were weakly and significantly correlated to one another. The maximum correlation was observed between perceived efficacy and cues to action (*r* = 0.394, *p* < 0.001) (Table 4).

**Table 4:** Pearson’s correlation coefficients (r) for practicing HIV prevention behavior among FSWs in Dima district, Southwest Ethiopia, 2019 (*N* = 449)

Variables	1	2	3	4	5	6	7
Prevention behavior	1						
Perceived susceptibility	-.035	1					
Perceived severity	-.070	.372^a^	1				
Perceived benefit	.020	.185^a^	.211^a^	1			
Perceived barrier	-.147^**^	.222^a^	.298^a^	.056	1		
Perceived efficacy	.097^*^	.163^a^	.232^a^	.425^a^	.158^a^	1	
Cues to action	-.012	.189^a^	.301^a^	.383^a^	.189^a^	.394^a^	1

^a^Correlation is significant at the 0.01 level (2-tailed)

^b^Correlation is significant at the 0.05 level (2-tailed)

### Factors associated with HIV prevention behavior

Multivariable binary logistic regression analysis was conducted with variables that demonstrated a *p*-value of less than 0.25 during simple binary logistic regression. Accordingly, the age of the respondent was the only socio-demographic factor significantly associated with HIV prevention behavior. Though it was found to be significant in bivariate analysis, marital status became insignificant after adjusting for possible confounders. Similarly, Knowledge about HIV and attitude towards HIV prevention methods were other variables significantly associated with HIV prevention behavior. Furthermore, self-efficacy and perceived barriers were the only HBM constructs significantly associated with HIV prevention behavior (*p*-value < 0.05) (Table 5).

**Table 5:** Factors associated with HIV prevention behavior among FSWs in Dima district, Southwest Ethiopia, 2019 (*N* = 449)

Variables	Category	HIV prevention behavior		COR(95%CI)	*p*-value	AOR(95%CI)	*p*-value
		high	low				
Age	16–19	115	64	1			
	20–24	98	67	.814(.526,1.259)	.355		
	> = 25	78	27	1.608(.943,2.742)	.081	1.911(1.100,3.320)	.022**
Marital status	In union^a^	48	17	1.638(.907,2.958)	.101		
	Single	243	141	1			
Knowledge of HIV	low	92	70	1			
	high	199	88	1.721(1.154,2.566)	.008	1.632(1.083,2.458)	.019**
Attitude toward HIV Prevention methods	Unfavorable	168	57	2.420(1.623,3.608)	.000	2.335(1.547,3.523)	.000***
	Favorable	123	101	1			
Perceived barrier	Low	118	81	.648(.439,.958)	.029	.627(.423,.930)	.020**
	High	173	77	1			
Self-efficacy	Low	144	97	1			
	High	147	61	1.623(1.094,2.408)	.016	1.667(1.107,2.511)	.014**

^**^Significant at *p* < 0.05

^***^Significant at *p* < 0.001

^a^Married, Divorced, widowed and separated

Female sex workers whose age was ≥ 25 years were 1.911 times more likely to engag in HIV prevention behavior as compared with those whose age was 16–19 (AOR = 1.911,95% CI: 1.100, 3.320). The likelihood of being engaged in HIV prevention behavior was 1.632 times higher among FSWs having a high knowledge of HIV compared to their counterpart (AOR = 1.632, 95% CI: 1.083, 2.458). The odds of engaging in HIV prevention behavior among those who had a favorable attitude towards HIV prevention methods were 2.335 times higher compared to those who did not have a favorable attitude toward the methods (AOR = 2.335, 95% CI: 1.547,3.523).

Similarly, being engaged in HIV prevention behavior was 37.30% less likely among FSWs who had high perceived barriers to HIV prevention methods compared to those who had low perceived barriers (AOR = 0.627, 95% CI: 0.423,0.930). Also, respondents who had high self-efficacy in HIV prevention methods were 1.667 more likely to practice HIV prevention methods than those with low self-efficacy (AOR = 1.667, 95% CI: 1.107, 2.511).

## Discussion

The international community planned a strategy of 'Ending inequality' for 'Ending the AIDS epidemic' by 2030, with considerable attention to the key population such as Female sex workers [1, 2]. However, they are given little attention in developing countries including Ethiopia [2, 7]. This study aimed at assessing HIV prevention behavior and associated factors among FSWs in Dima district, Gambella regional state of Ethiopia; using HBM.

Accordingly, the study revealed that about two third (64.8%) of FSWs had high HIV prevention behaviors. This is in line with a similar study conducted in the Afar region [18] and Dire Dawa [22], Indonesia [23]. The similarity of these findings could be due to the closeness of socio-culture, the way of living of the FSWs, and the nature of the work [24]. However, this figure is greater than the cross-sectional studies conducted in northern parts of Ethiopia [19, 25]. This may be due to some sort of intervention in the region that may enable this key population to follow the aforementioned HIV prevention methods [6, 7]. Yet, this magnitude showed the need to emphasize this population for achieving the ambitious goal of 'Ending inequality' for 'Ending the AIDS epidemic' by 2030 [1, 2].

Moreover, age, knowledge of HIV, attitude toward HIV prevention methods, perceived barriers, and self-efficacy were identified as having statistical associations with HIV prevention behavior among FSWs. Specifically, female sex workers whose age was ≥ 25 years were more likely to engage in HIV prevention behavior as compared with those whose age ranges 16–19. Our finding is comparable with the result of previous studies conducted in northern parts of Ethiopia [19, 25] and the report of the United Nations Program on HIV/AIDS (UNAIDS) [26]. This might be attributed by fearing of discrimination against service-seeking among FSWs in the lower age group [27, 28]. This could also be caused by a lack of knowledge about HIV due to young age [29]. Consequently, they remain reluctant to consistent condom usage and testing for HIV [1, 26, 30].

In support of this, a qualitative study conducted in a similar setting disclosed that youths conduct sex in “Tifo bet” (residential youth accommodation, where young people may interact with mineworkers and other sexual partners) to keep their sexual relations secret from the witness of people; without using a condom or knowing the HIV status of their sexual partners; in exchange for small things like soap, lotions, or money [11]. This finding implies the importance of designing differentiated responses that meet the needs of adolescent FSWs.

In addition, HIV prevention behavior was higher among FSWs having a high knowledge of HIV as compared with their counterparts. This finding is consistent with similar studies conducted in different parts of Ethiopia [18, 19, 22, 25] and Indonesia [23]. This could be due to the fact that individuals who know the transmission and prevention pathway can strictly follow the recommended prevention practice including consistent condom use, testing for HIV, and not sharing injecting equipment with another person. However, a qualitative study conducted in a similar setting explained the shifting of government and NGOs working on HIV to other programs as contributing factors for sustained HIV in the area [11]. This may call for reconsidering the program for solidification of HIV prevention.

Together with these, the odds of practicing HIV prevention methods were higher among FSWs with favorable attitude toward the aforementioned HIV prevention methods as compared to their counterpart. Supporting this, similar studies conducted inDire Dawa [22] and Indonesia [23] revealed low HIV prevention practices among FSWs with a negative attitude toward the HIV prevention methods. This might be due to discrimination and rude remarks from providers, denial or delay of services, and potential for breach of confidentiality. These may be revealed well by a quote from Uganda which disclosed, *“When they know that you are a sex worker, you will be the last person to be treated”* [31].

Furthermore, FSWs who had high self-efficacy in practicing the recommended HIV prevention behavior were more likely practice HIV prevention behavior as compared with those who had low self-efficacy. This is in line with research findings from Ethiopia [32], Ghana [21], and the report of United Nations Program on HIV/AIDS (UNAIDS) [26]. This might be due to the pivotal role of self-efficacy in building an individual’s belief about his/her ability to practice the recommended prevention behavior [13]. This may call for the need for further capacitating FSWs about their confidence in conducting the aforementioned HIV prevention practice.

Lastly, odd of HIV prevention behavior were less likely to be implemented among FSWs who have high perceived barriers to prevention methods compared with those with low perceived barriers. This is consistent with the study conducted in Ghana [21]. In support of this result; the cognitive-behavioral theories indicated that people with high perceived physical or psychological barriers or inconveniences and unpleasantness to certain behavior are more skeptical about performing the behavior [12]. This may imply the need for friendly service provision for these population segments.

In this study, perceived barriers and self-efficacy were the only HBM constructs found to be significantly associated with HIV prevention behavior. This might be related to the fact that all constructs of HBM may not necessarily predict a range of behaviors with health implications [12, 13]. Such variations in number and type of construct affecting the prevention methods are also observed in similar studies conducted using the model [14–16]. In a cross-sectional study conducted in Gambella town using the model, for instance, perceived severity of HIV and perceived benefit of engaging in HIV prevention behavior were the only two constructs of the model significantly associated with the recommended prevention methods [14].

These differences might be related to the construction of the outcome variable. In this study; the outcome variable was constructed by combining different categories of prevention methods. However; many other studies used individual components in their studies [14–16]. It might also be due to relatively high knowledge of FSWs about HIV in general and transmission pathways, severity, and benefit of the recommended HIV prevention methods, in particular, in this era of the information. As a result, what could matter is about hindering factors against practicing the recommended HIV prevention methods and their competence in doing so. This may call for designing strategies for endorsing FSWs’ efficacy and managing perceived barriers so that they can practice the recommended HIV prevention methods.

### Strength and limitation

This study has several strengths. The study is based on a theoretical framework guided by the Health Belief Model, the most recommended model for forecasting why people do not take preventive measures for health promotion and disease prevention [12, 13, 21]. In addition, the study considered other variables beyond the HBM constructs, including knowledge of HIV/AIDS and attitude toward HIV prevention methods.

However, the study is not without limitations. The study participants were sampled by the snowball sampling technique, which may affect the generalizability of the finding to the true population. To resolve this, we make the study community-based so that it can represent the true population. Since the study was conducted using self-reported data, it might also be subjected to the social desirability bias that may overestimate the findings. However; some studies have shown a correspondence between the self-report data and the observational data [33, 34]. Furthermore, the current study did not identify participants as HIV-positive or negative individuals. Therefore, the findings of this study should be interpreted for all FSWs in the study area, without their sero status.

## Conclusion

The study identified that about two-thirds (64.8%) of FSWs had high HIV prevention behavior. The age of the respondent, knowledge of HIV, and attitude toward the recommended HIV prevention method was found to have a statistically significant relationship with HIV prevention behavior. However, self-efficacy and perceived barriers were the only HBM constructs found to be significantly associated with HIV prevention behavior. Therefore, focusing on these factors would be instrumental for improving the effectiveness of the ongoing HIV prevention efforts and attaining the "Sustainable Development Goals" of "ending inequality" and "ending the AIDS epidemic" by 2030.

## Data Availability

The datasets presented in this article are not readily available because of privacy and ethical concerns. However, reasonable requests to access the datasets should be directed to gachanamidaksa24@gmail.com.

## Abbreviations

AIDS: Acquired immunodeficiency syndrome
FSWs: Female Sex Workers
HBM: Health Belief Model
HIV: Human Immune virus
SD: Standard Deviation
SPSS: Statistical Package for Social science
SDG: Sustainable Development Goal
UNAIDS: The joint United Nations Programmes on HIV/AIDS
